# Gastric Conduit Perforation: A Late Fatal Complication after Esophagectomy

**DOI:** 10.7759/cureus.4987

**Published:** 2019-06-24

**Authors:** Aditya A Kulkarni, Vivek Chauhan, Vishal Sharma, Harjeet Singh

**Affiliations:** 1 Department of General Surgery, Postgraduate Institute of Medical Education and Research (PGIMER), Chandigarh, IND; 2 Department of Gastroenterology, Postgraduate Institute of Medical Education and Research (PGIMER), Chandigarh, IND

**Keywords:** surgery, emergency, cancer, infection, esophagus

## Abstract

With an increasing number of long-term survivors of carcinoma esophagus, it is important to be vigilant about postoperative complications related to gastric tubes. Perforation of the gastric conduit has been rarely seen, with very few case reports in the literature. We report a rare case of perforation of the gastric tube conduit in a patient who had previously undergone esophagectomy for squamous cell carcinoma of esophagus five years ago. The patient presented with diffuse peritonitis in an emergency. On exploration, a large perforation was present on the anterior wall of the gastric conduit. This was closed with primary suture repair. Histopathology revealed nonspecific inflammatory changes. Unfortunately, the patient succumbed to severe sepsis and multiorgan dysfunction despite early surgical intervention and critical care management. Conduit perforation can be a major source of morbidity and mortality. Although gastric conduit is predisposed to ulcer formation due to multiple reasons, conduit perforation may occur only in rare cases. We recommend that surveillance endoscopy for conduit ulcer should be performed in long-term survivors after esophagectomy. Patients with conduit ulcers would benefit from long-term acid suppression with proton-pump inhibitors.

## Introduction

In recent years, there has been an improvement in the prognosis of patients with carcinoma esophagus. Five-year overall survival rates are reported to be as high as 30% to 40% in patients who undergo resection [1]. Surgical resection i.e esophagectomy is the treatment of choice for patients who present with resectable disease. Reconstruction following esophagectomy can be done in a number of ways (gastric, colonic or jejunal conduits). The vast majority of reconstructions after resection for esophageal cancer are performed with the tubularized stomach as a conduit [1]. With better survival of these patients, complications related to the gastric tube, such as ulcer formation, may be seen. Perforation of a gastric tube ulcer is a rare and catastrophic complication. Here, we report a case of gastric tube ulcer perforation into the peritoneal cavity five years after esophagectomy for carcinoma esophagus.

## Case presentation

A 62-year-old man presented to the emergency surgery outpatient department with complaints of acute severe abdominal pain for the past two days. Physical examination was remarkable for tachycardia, hypotension, and marked abdominal guarding and rigidity. He had a past history of squamous cell carcinoma of the lower thoracic esophagus, for which the patient underwent transhiatal esophagectomy with orthotopic gastric tube conduit five years ago. The patient was a chronic smoker for many years, and also gave a history of occasional alcohol use. Laboratory studies revealed leucocytosis (white cell count 13,500), severe metabolic acidosis (pH=7.19) and deranged renal parameters with severe hyperkalemia (Sodium-133 meq/L, Potassium-5.8 meq/L, Urea-138 mg/dL, Creatinine 4.12 mg/dL). 

Ultrasound of the abdomen showed gross ascites with multiple septations. Erect X-ray abdomen revealed free air under the diaphragm, confirming the diagnosis of perforation peritonitis. The patient was resuscitated with intravenous fluids and antibiotics. Emergency dialysis was performed in view of refractory hyperkalemia and severe metabolic acidosis. Bilateral flank drains were inserted with a view to draining out the infected fluid in order to attain partial source control. These drained around 1000 ml of foul smelling greenish fluid. 

**Figure 1:**
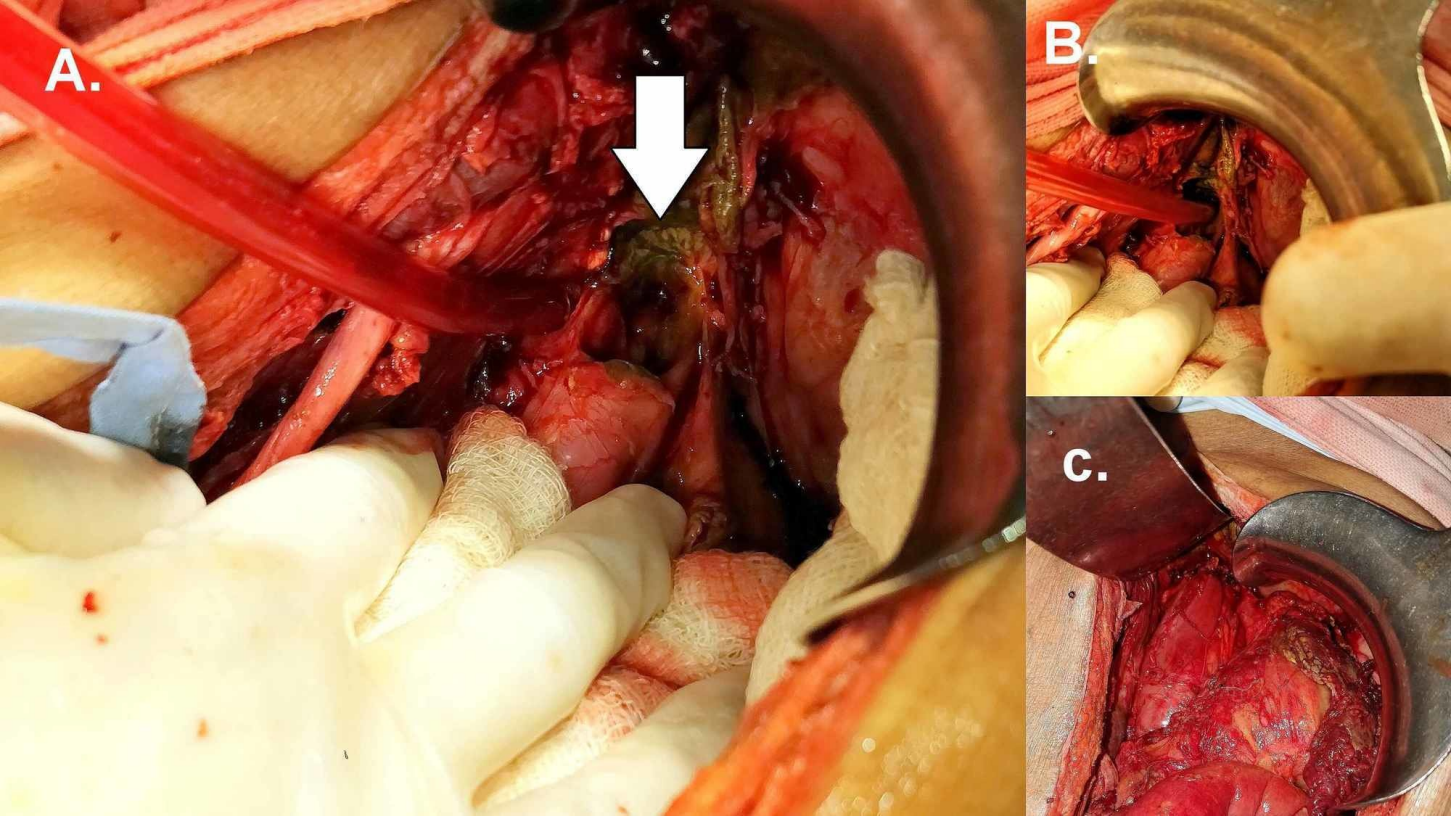
Operative photograph 1A and 1B. Perforation of the gastric tube seen at the hiatus (1C). After primary repair of the perforation.

He responded favorably to initial resuscitation and was taken up for emergency surgery. At laparotomy, there was gross intraperitoneal contamination with biliopurulent fluid. There were dense inter-bowel and parietal adhesions. After extensive adhesiolysis, a large perforation measuring 3 cm by 3 cm was found on the anterior aspect of the distal part of the gastric tube conduit, approximately 2 cm proximal to the pylorus [Figure 1a and 1b]. The perforation site was immediately below the right crus, which was partially covering the perforation site. The edges were edematous and friable. After thorough peritoneal toileting, the perforation was closed primarily after freshening the margins [Figure 1c]. Witzel-type feeding jejunostomy was placed to ensure enteral nutrition in the postoperative period. Postoperatively, he remained intubated and on inotropic support in view of severe sepsis and shock. Dialysis was repeated on postoperative day one. However, he failed to respond to supportive management and succumbed to his illness on postoperative day two. Histopathologic examination of biopsy from perforation edge revealed non-specific inflammation.

## Discussion

Tubularized stomach conduit with cervical esophagogastric anastomosis is the commonest method of reconstruction after esophagectomy for esophageal carcinoma [1]. Motoyama et al. in a prospective study reported a 47% prevalence of secondary gastric tube disorders [2]. Prevalence of ulcer formation in the gastric tube ranges from 6.1%- 19.4% [2-4]. Although ulcer formation is common, perforation of a gastric tube ulcer is a very rare complication, with one review revealing only thirteen cases in the English literature [5].

Various causes and mechanisms of gastric tube ulceration have been proposed. Acid production in the gastric tube is expected to be low considering the loss of acid-secreting mucosa and vagotomy as a part of the surgical procedure. However, gastric acid secretion recovers with time and can return to normal [6-7]. This may predispose to ulcer formation. Blood supply to the gastric tube, especially in the proximal lesser curve is tenuous. Hence ulcer formation is common in this location [5]. However, the perforation in our case was in the prepyloric region, which raises the possibility of other causative factors. Excessive regurgitation of alkaline duodenal contents is injurious to the gastric mucosa and may promote ulceration and impair ulcer healing, especially with posterior mediastinal reconstruction like in our case [8]. Tumour recurrence, radiation, use of non-steroidal anti-inflammatory drugs and gastric stasis secondary to denervation are other putative causes.

Route of reconstruction may play a part in predisposing to ulcer formation. Aiko et al. reported an increased incidence of peptic ulcers in the gastric tube reconstructed via the retrosternal route as compared to the posterior mediastinal route [9]. The average duration from esophagectomy to ulcer perforation has been reported as three years and four months [5], but cases have been reported as late as 12.5 years after surgery [10]. Mortality after perforation of tube ulcer is reported to be as high as 84.6% [5]. Conduit ulcer may perforate into various locations like aorta, trachea, bronchus, thoracic cavity, pericardium or sternum [5, 11]. However, our case was different in that there was free perforation into the peritoneal cavity. To the best of our knowledge and literature review, this is the first report of such complication after esophagectomy. Due to the rarity of this complication, it is difficult to suggest an ideal approach to its management. Laparotomy with either primary repair or omental patch closure is the best possible option in a patient with peritonitis, as in the index case. A high index of suspicion and early diagnosis and treatment are critical to ensure a successful outcome. It is especially important to be vigilant about surveillance and treatment of gastric tube ulcers, given the significant incidence of ulceration and high mortality after perforation. More than 50% of gastric tube ulcers do not cause pain, possibly as a result of vagotomy [12]. Hence, a surveillance program may be warranted in post-esophagectomy patients, especially those at risk (smoker, NSAID user). In patients who are detected to have conduit ulcers, detection, and treatment of *Helicobacter pylori* and acid suppression with proton pump inhibitors may be done, to minimize the chance of ulcer complication.

## Conclusions

This case illustrates a rare and unprecedented scenario of intraperitoneal perforation of a gastric conduit ulcer. This is a serious complication with high morbidity and mortality. Gastric conduit is prone to develop ulcers for various reasons. Periodic endoscopic surveillance of the conduit for ulcers and early detection and treatment of *H. pylori *is essential to prevent this complication. 
